# The TCR repertoire of α-synuclein-specific T cells in Parkinson’s disease is surprisingly diverse

**DOI:** 10.1038/s41598-020-79726-9

**Published:** 2021-01-11

**Authors:** Akul Singhania, John Pham, Rekha Dhanwani, April Frazier, Juliana Rezende Dutra, Karen S. Marder, Elizabeth Phillips, Simon Mallal, Amy W. Amara, David G. Standaert, David Sulzer, Bjoern Peters, Alessandro Sette, Cecilia S. Lindestam Arlehamn

**Affiliations:** 1 Division of Vaccine Discovery, La Jolla Institute for Immunology, La Jolla, CA 92037 USA; 2 Department of Neurology, Columbia University Medical Center, New York, NY 10032 USA; 3 Institute for Immunology and Infectious Diseases, Murdoch University, Perth, WA 6150 Australia; 4 Vanderbilt University School of Medicine, Nashville, TN 37235 USA; 5 Department of Neurology, University of Alabama at Birmingham, Birmingham, AL 35233 USA; 6 Department of Psychiatry, Columbia University Medical Center, New York, NY 10032 USA; 7 Department of Pharmacology, Columbia University Medical Center, New York, NY 10032 USA; 8 Department of Medicine, University of California San Diego, La Jolla, CA 92093 USA

**Keywords:** Immunology, Adaptive immunity, Autoimmunity

## Abstract

The self-antigen α-synuclein (α-syn) was recently shown to be associated with Parkinson’s disease (PD). Here we mapped the T cell receptor (TCR) repertoire of α-syn-specific T cells from six PD patients. The self-antigen α-syn-specific repertoire was compared to the repertoire of T cells specific for pertussis (PT), as a representative foreign antigen that most individuals are exposed to, revealing that the repertoire for α-syn was as diverse as the repertoire for PT. The diversity of PT-specific clonotypes was similar between individuals with PD diagnosis and age-matched healthy controls. We found that the TCR repertoire was specific to each PD patient, and no shared TCRs among patients were defined, likely due to differences in HLA expression that select for different subsets of epitope-specific TCR rearrangements. This study provides the first characterization of α-syn-specific TCR clonotypes in individuals with PD. Antigen-specific TCRs can serve as immunotherapeutics and diagnostics, and means to track longitudinal changes in specific T cells, and disease progression.

## Introduction

Recognition of T cell epitopes is dependent on the expression of specific HLA molecules that bind the peptides and the presence of T cells expressing specific T cell receptors (TCRs). TCRs are generated by somatic recombination during lineage development. The theoretically possible combinatorial diversity of the TCR repertoire has been estimated to be > 10^15^ distinct αβ receptors or clonotypes^1^, which is many more sequence combinations than the number of T cells in an individual^2^. The actual repertoire that emerges after the processes of positive and negative selection is much more restricted, as many of the potentially autoreactive TCRs are eliminated^3^. We and others have shown that the presence of a precursor repertoire of naïve T cells capable of recognizing an epitope is a good predictor of the magnitude of response against an epitope^4–7^. The CDR3 region of the TCR-β chain is the most polymorphic and makes direct contact with the epitope, and sequencing of this region is sufficient to generate a marker of epitope-specific T cells.

In humans, the development of immune responses in infection, vaccination, and disease results in the selective expansion of specific T cells^3^. In the setting of autoimmune disease and non-mutated cancer antigens, the TCR repertoire of epitope-reactive T cells is thought to be significantly less diverse^8^, as high affinity TCR clones are thought to be eliminated and inactivated by thymic education, as well as central and peripheral tolerance^3^.

We previously described that individuals with Parkinson’s disease (PD) possess T cells that recognize specific epitopes derived from the PD associated protein α-synuclein (α-syn)^9^, indicating the presence of autoimmune features in this disease. Recently, we found that α-syn-reactive T cells are most abundant immediately after diagnosis of motor PD and may be present years before the diagnosis of motor PD^10^. In contrast to the case of PT, and in accordance with the notion that the autoimmune repertoire is narrowed by thymic selection, the responses to α-syn are weaker, requiring an in vitro amplification step to be detected. Moreover, this is consistent with the relatively small size of α-syn, which contains two main epitope regions^9^.

Here we studied the TCR repertoire of α-syn T cells from PD patients and compared it to the repertoire of T cells specific for PT (aP vaccine antigens) as a control. We further examined whether the increased frequency of T cells responding to specific α-syn epitopes in PD subjects is associated with the presence of shared ‘public’ TCRs in PD patients recognizing these epitopes. As expected based on the wide diversity of HLA that select for different subsets of epitope specific TCR rearrangements in the population, we found that the α-syn-specific TCR repertoire differed among PD patients. We did not find any public TCRs, but rather we found that the TCR repertoire directed to the self-antigen α-syn was as diverse as the TCR repertoire directed to the foreign PT antigens.

## Results

### Similar numbers of clonotypes expand upon stimulation with α-syn and PT peptide pools

**Figure 1:**
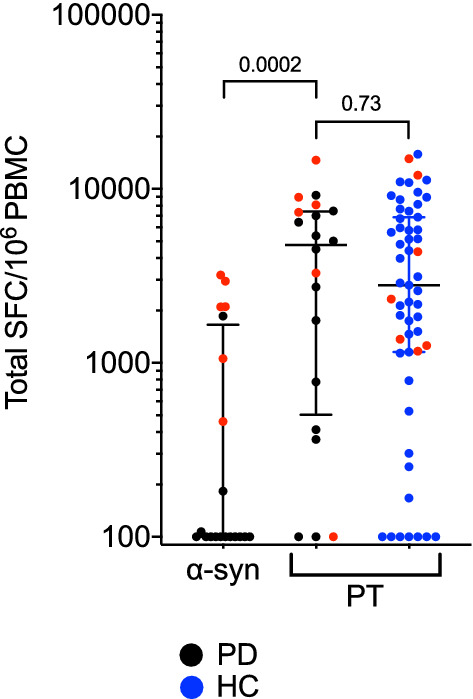
Magnitude of responses against α-syn and PT peptides. Magnitude of responses (sum of IFNγ, IL-5, and IL-10) against α-syn and PT peptides as SFC per 10^6^ cultured PBMC (14-day in vitro expansion). Each point and symbol represents one participant. Red symbols indicate participants that were selected for TCR sequencing. Median ± interquartile range is shown.

We have previously described the identification of α-syn and PT reactive T cells^9,11^. Briefly, to detect relatively rare antigen-specific CD4 T cells, like α-syn-specific T cells, PBMCs were stimulated for 14 days in vitro with epitope pools for α-syn or PT. After two weeks, cultures were harvested and stimulated with epitope pools, and analyzed for cytokine production in a triple-color IFNγ, IL-5 and IL-10 Fluorospot assay. In this study we tested 20 individuals with PD for T cell reactivity against α-syn and PT, as well as 55 age-matched HC against PT (Fig. 1). We assumed that the vast majority of individuals in our cohort above 45 years of age will have been exposed to or vaccinated against PT. As expected, 65/75 individuals responded to the PT peptide pool. Additionally, the magnitude of response against PT was significantly higher (two-tailed Mann–Whitney *p* = 0.0002) than that for α-syn-specific responses (Fig. 1). No difference was observed in the magnitude of response against PT when comparing PD with age-matched HC (two-tailed Mann–Whitney *p* = 0.73) (Fig. 1). HC were not tested for α-syn reactivity, as the α-syn reactivity is specific for PD, and HC react only occasionally and with significantly lower magnitude^9^.

**Figure 2:**
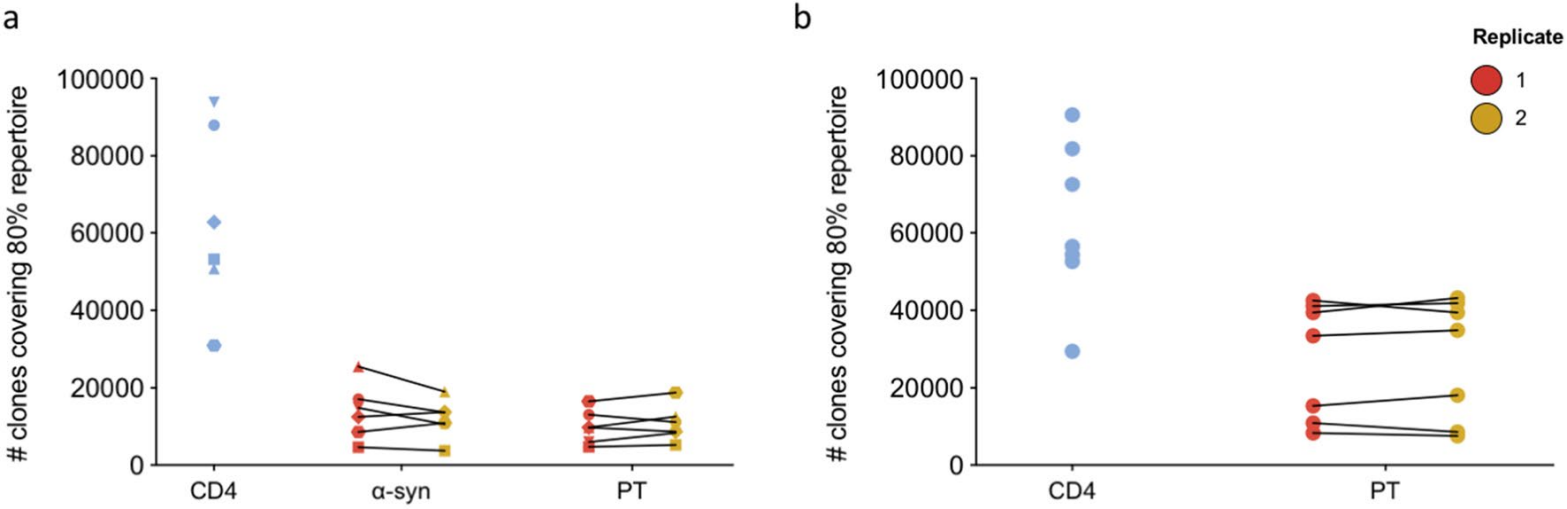
Similar number of clonotypes expand in response to α-syn and PT peptide pools. Number of TCR clonotypes covering 80% of the productive repertoire. Ex vivo CD4 repertoire (blue) captured immediately following thaw. Two replicates (red and yellow) were sequenced for each peptide pool stimulation (14-day in vitro expansion). Each point and symbol represents one participant. **a** PD n = 6, **b** HC n = 7.

For TCR sequencing, we selected 6 of the individuals with PD who had α-syn-specific T cell reactivity as determined by the Fluorospot assay (Fig. 1, red symbols; Table 1) and 7 age-, sex-, and recruitment site-matched HC (Fig. 1, red symbols; Table 1). PBMC cultures, 2 million PBMCs were cultured per replicate, were harvested and DNA was purified for TCR sequencing using the ImmunoSEQ service from Adaptive Biotechnologies. Each sample included a culture replicate and as a comparison, the ex vivo repertoire of CD4 T cells was determined. The productive repertoire of each sample, i.e., the unique in-frame rearrangements that do not contain a stop codon, and the frequency of these productive clonotypes within the sample was assessed. The number of clonotypes covering eighty percent of the productive repertoire was similar between replicates of the same sample (Fig. 2a, b). In PD, the number of clonotypes contributing to the productive repertoire was similar upon stimulation with either the α-syn or PT peptide pools (Fig. 2a). In HC, however, a wider number of clonotypes covering eighty percent of the productive repertoire following stimulation with PT peptides (Fig. 2b). As expected, all donors exhibited a decrease in the number of productive rearrangements as a result of stimulation when compared to the ex vivo samples (Fig. 2a, b), suggesting the selection and expansion of specific clonotypes upon stimulation with peptide pools, leading to fewer unique rearrangements within the sample.

**Table 1:** Summary of demographic characteristics of enrolled participants.

	Total cohort	Individual PD participants accessed by TCR sequencing						Total cohort	Individual HC participants accessed by TCR sequencing						
Characteristics	PD	3450	3460	3486	3489	3529	3530	HC	3447	3452	3457	3459	3466	3487	3495
Participants enrolled, n	20							55							
Age, median, range, years	58.5 (51–73)	58	51	73	62	55	57	66 (53–92)	56	53	55	60	70	69	57
Sex, male, % (n)	60 (12)	M	F	F	M	M	F	49 (27)	F	F	M	M	M	F	M
Caucasian, % (n)	95 (19)	White	White	White	White	White	Asian	93 (51)	White	White	White	White	White	White	White
Age at diagnosis of PD, median, range, yr	55.5 (48–72)	54	50	72	60	54	57	N/A	–	–	–	–	–	–	–
Years since diagnosis, median, range, yr	1 (0–4)	4	1	1	2	1	0	N/A	–	–	–	–	–	–	–
UPDRS, median, range	24.5 (12–37)	28	12	23	37	26	16	N/A	–	–	–	–	–	–	–
MoCA, median, range	25.5 (21–28)	28	27	28	21	24	23	N/A	–	–	–	–	–	–	–
LED, median, range	250 (0–450)	400	450	0	450	100	0	N/A	–	–	–	–	–	–	–
Total SFC (α-syn, sum of IFNγ, IL-5 and IL-10)	–	1057	2950	460	2097	2103	3200	–	–	–	–	–	–	–	–
Total SFC (PT, sum of IFNγ, IL-5 and IL-10)	–	100	3278	8947	14,620	7330	8087	–	1167	1367	2323	4350	14,900	1257	11,987

### Stimulation with α-syn and PT leads to the expansion of specific clonotypes

We next explored the repertoire of α-syn- or PT-specific clonotypes in our samples. To this end, we compared the productive repertoire of samples stimulated with the peptide pools to the corresponding ex vivo CD4 samples, and identified clonotypes that were significantly perturbed as a result of either α-syn or PT stimulation (Table 2 and Supplementary Fig. 1). As expected, of the clonotypes that were significantly perturbed upon stimulation, the majority expanded and had higher frequencies compared to the ex vivo CD4 samples (Supplementary Fig. 1).

**Table 2:** Clonotypes expanded upon stimulation with α-syn or PT peptides.

Specificity	Cohort	Participant	Total no. of clonotypes (replicate 1/2)	No. of expanded clonotypes (replicate 1/2)	No. of expanded clonotypes in both replicates (% of expanded repl. 1/2)	No. of antigen-specific clonotypes (% of expanded in both repl.)
α-syn	PD	3450	25,885/21,702	231/278	56 (24/20)	16 (29)
		3460	8772/5497	124/67	19 (15/28)	5 (26)
		3486	36,306/25,617	190/122	48 (25/39)	15 (31)
		3489	19,383/20,466	199/197	67 (34/34)	17 (25)
		3529	13,444/16,356	136/137	33 (24/24)	9 (27)
		3530	24,402/17,858	225/204	49 (22/24)	13 (27)
PT		3450	23,999/20,713	287/226	61 (21/27)	23 (38)
		3460	6695/7273	78/70	17 (22/24)	3 (18)
		3486	12,934/16,804	93/108	30 (32/28)	5 (17)
		3489	16,772/18,366	258/264	75 (29/28)	20 (27)
		3529	24,283/27,116	122/135	48 (39/36)	17 (35)
		3530	12,932/16,636	269/319	47 (17/15)	17 (36)
PT	HC	3447	65,296/64,818	135/134	56 (41/42)	–
		3452	60,026/64,994	270/239	45 (17/19)	–
		3457	56,571/58,480	299/363	121 (40/33)	–
		3459	37,002/41,917	194/143	78 (40/55)	–
		3466	30,810/29,033	241/221	112 (46/51)	–
		3487	64,542/61,252	123/127	50 (41/39)	–
		3495	28,371/26,341	122/182	37 (30/20)	–

**Figure 3:**
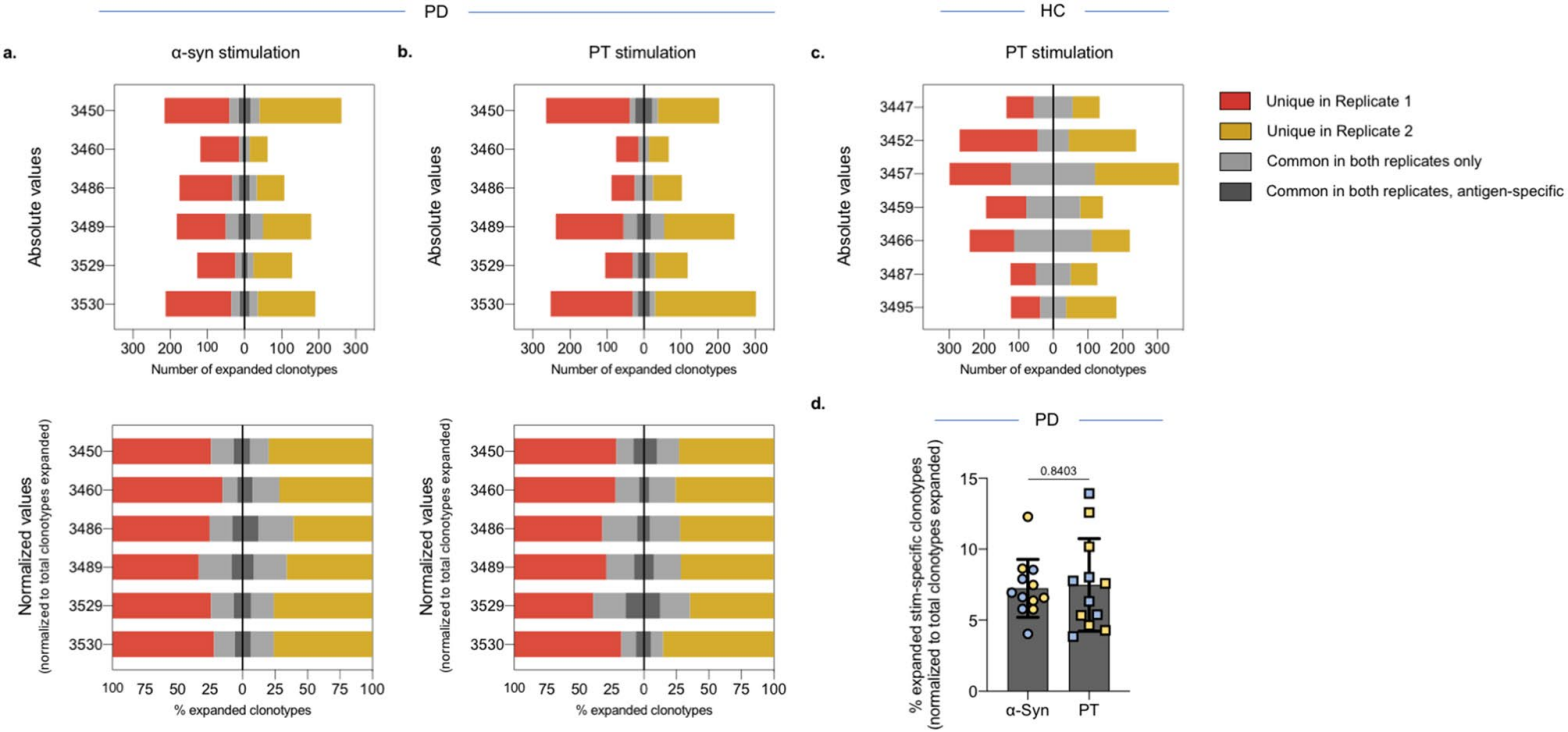
Similar numbers of expanded clonotypes in response to α-syn and PT stimulation. The number of expanded clonotypes in α-syn- and PT-stimulated PD (**a**, **b**) and HC (**c**) samples. For PD both absolute numbers (top) and normalized against total number of clonotypes in each replicate (bottom) is shown. Red indicates clonotypes that are unique in replicate 1, yellow indicates clonotypes that are unique in replicate 2. Grey indicates clonotypes that are common between the two replicates (− log_2_ OR > 1 and FDR *p* val < 0.05) and dark grey indicates antigen-specific clonotypes, i.e., number and proportion of clonotypes that expanded only in each antigen-stimulated culture excluding clonotypes that expanded irrespective of stimuli. **d** Percentage of α-syn- and PT specific clonotypes in PD (dark grey in bottom graphs). Yellow (replicate 1) and blue (replicate 2). Mean ± StDev is shown. Paired two-tailed *t* test.

In our assays, we included replicate samples to allow identification of the clonotypes that reproducibly expanded within a single donor. Similar numbers of clonotypes were significantly expanded in each of the replicate samples from a donor (− log_2_ OR > 1 and FDR *p* val < 0.05 in both replicates; Table 2, Fig. 3a–c, and Supplementary Fig. 1). For α-syn stimulated samples an average of 176 clonotypes per replicate was found. In the case of PT stimulated samples, we found similar number of expanded clonotypes per replicate when comparing the two different cohorts, with an average of n = 186 clonotypes per replicate in PD and an average of n = 200 in HC. On average, the common clonotypes between the replicates corresponded to 30% of the total expanded clonotypes in the donor (ranging from 15 to 55%; Table 2, Fig. 3a–c).

### Stimulation with α-syn or PT results in participant-specific TCR expansion

Having determined the overlap between replicates in in vitro stimulated cultures, we next investigated whether repertoire overlap was detected between the 6 different PD subjects or the 7 different HC subjects. Based on the exact clonotype rearrangement sequence, none of the clonotypes identified by α-syn or PT stimulation were observed across all individuals, suggesting that the various subjects are associated with different individual repertoires (Supplementary Fig. 2).

We also assessed overlap between individuals using the GLIPH algorithm^12^, which clusters TCR sequences based on similarities in enrichment for either 3-/4-mers, convergence groups (a set of multiple TCRs from one or more individuals that bind the same antigen in a similar manner through similar TCR contacts), global convergence (a pair of TCRs that share the same length CDR3 and differ by less than 1 amino acid in those CDR3 regions), and local convergence (a pair of TCRs that share in their CDR3 regions an amino acid motif that appears enriched in their sample set). The GLIPH analysis revealed that there was higher similarity between replicate samples within a single individual and essentially no overlap across individuals (Supplementary Table 1). The general lack of overlap also reflects the HLA class II allele diversity in the test population (Supplementary Table 2).

### Definition of α-syn- or PT-specific clonotypes

To capture clonotypes that were specifically altered as a result of α-syn stimulation and not due to effects associated with non-specific bystander activation in in vitro culture, we compared the clonotypes significantly expanded in response to α-syn stimulation with those that significantly expanded following PT stimulation in the PD participants. We focused on reproducible clonotypes identified in both replicate samples following stimulation with α-syn (Table 2, Supplementary Fig. 1b). Of these reproducible clonotypes (Fig. 3a, light gray bars), approximately 30% (25–31%) were specifically altered upon α-syn stimulation and not by PT stimulation (Fig. 3a, dark gray bars, Supplementary Fig. 1c).

**Figure 4:**
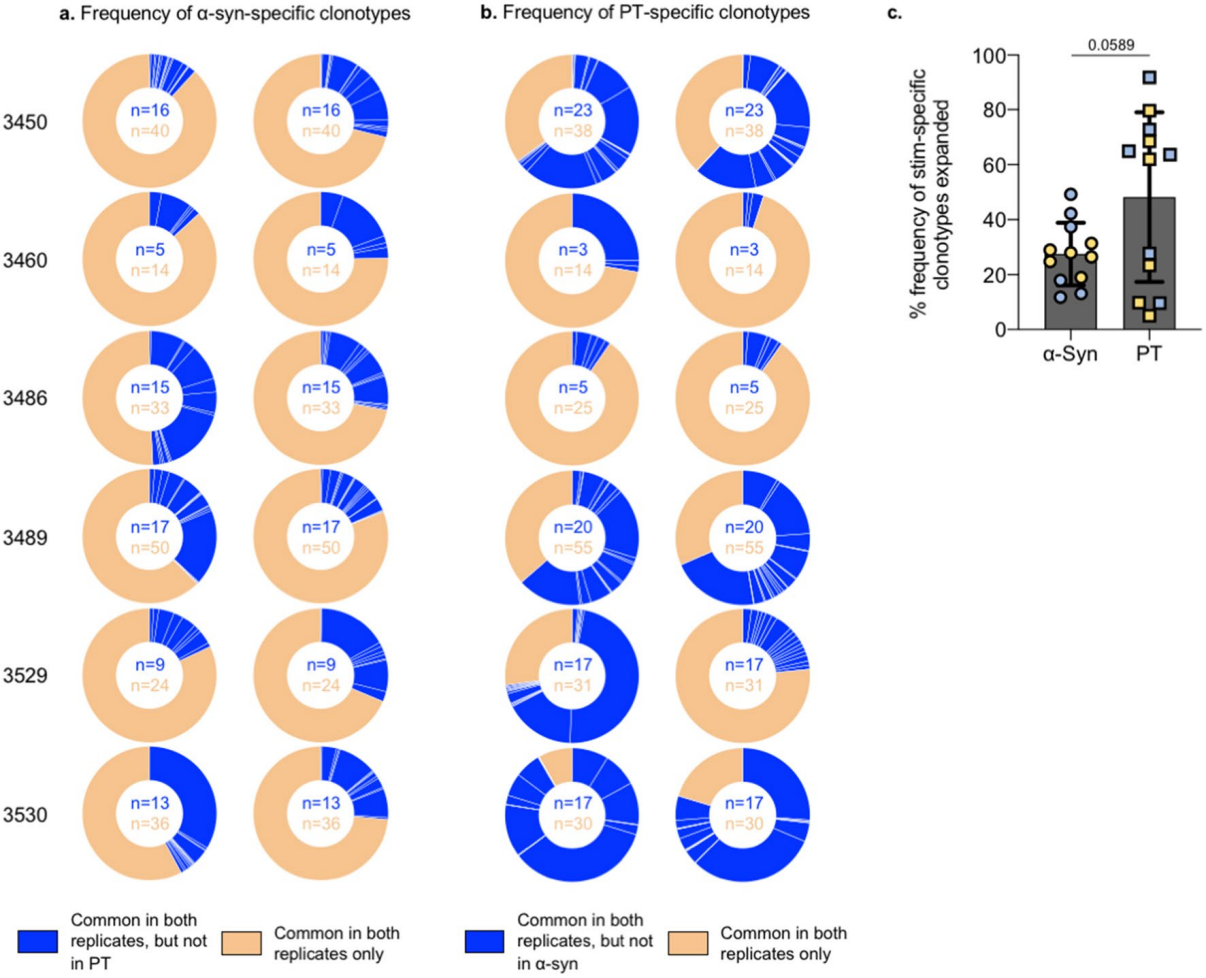
α-Syn-specific clonotypes have a similar frequency to the overall expanded clonotypes. Frequency of clonotypes expanded upon α-syn (**a**) or PT (**b**) stimulation in both replicates (excluding non-specific expansion in response to both stimuli conditions) in the total productive repertoire (normalized to respective combined total) in individual PD participants. **c** Percentage frequency of the expanded antigen-specific clonotypes for α-syn and PT. Yellow and blue indicates individual replicates. Mean ± StDev is shown. Paired two-tailed t-test.

Next, we assessed whether these α-syn-specific clonotypes (Fig. 3a, dark gray bars) had a higher expansion indicating a larger selection, compared to those clonotypes that were also reproducible between the two replicates, but were non-specific. To assess this, we determined the total templates of each of the α-syn-specific clonotypes and whether these templates corresponded to a higher frequency in the overall reproducible clonotype pool identified in both replicates (α-syn-specific + non-specific). On average, α-syn-specific clonotypes (which made up 30% of the reproducible clonotype pool) had an overall frequency of 28%, and the non-specific clonotypes (which made up 70% of the reproducible clonotype pool) had a frequency of 72% (Fig. 4a). This indicates a similar expansion and selection between these two clonotype groups.

Similar to the α-syn expansion, following PT stimulation (Table 2, Supplementary Fig. 1b), approximately 30% (17–38%) of the clonotypes were specifically altered upon PT stimulation in both replicates within a donor (Fig. 3b, Supplementary Fig. 1c). There were no significant differences in the number of antigen-specific clonotypes expanded in response to α-syn or PT stimulation (Fig. 3d). However, in contrast to the α-syn-specific clonotypes, on average these PT-specific clonotypes made up 48% of the frequency of the total reproducible clonotype pool, suggesting a greater expansion and selection of these clonotypes compared to the non-specific clonotypes that had a frequency of 52% (Fig. 4b). There was a trend toward the specific clonotype expansion against α-syn being more limited than that against PT (Fig. 4c), which may reflect general differences in response to foreign and self-antigens. However, this increased expansion of PT-specific clonotypes compared to the α-syn-specific clonotypes was true for some individuals, but not all (Fig. 4c).

## Discussion

Here we report an analysis of PD patients in regard to the α-syn-specific TCR repertoire, an autoantigen recently associated with PD^9,13^ and its progression^10^. The rationale for this analysis originates from the fact that detection of TCRs specifically associated with PD might provide a diagnostic tool, could generate insights into disease pathogenesis, and pave the way for adoptive transfer applications.

In terms of the potential for TCR-based diagnostics, our study demonstrates that TCRs can be mapped to α-syn responses in PD patients. Our stringent selection criteria of only focusing on clonotypes present in replicate samples identifies the TCRs that are antigen-specific, and may not identify the complete antigen-specific repertoire within an individual. Previous studies^12,14^ have found that within an epitope-specific repertoire, a portion of the responding receptors cluster closely together based on shared motifs. To find shared TCR clonotypes between individuals, we used direct sequence comparison, and the GLIPH algorithm^12^ applied to the clonotypes identified in two replicates. We did not find evidence for shared TCR repertoires for either α-syn or PT in the subjects studied, similar to findings for individuals with MS^15^. There are limited studies on autoantigen-specific TCR repertoires. However, in a study by Eugster et al., where they investigated the TCR repertoire of GAD65 autoantigen-specific human CD4 T cells, they also found high diversity in the repertoire^16^. These findings are not surprising, as these subjects were not matched based on their HLA expression. The response to α-syn, like any other known human antigens, is mediated by multiple epitopes, so that different individuals typically respond to different epitopes, which are restricted by diverse HLA alleles. While shared epitope-specific receptor chains have been observed in individuals sharing the relevant HLA alleles^17,18^, this is relatively uncommon and does not appear to be the case for PD associated α-syn-specific TCRs. Thus, while the present study provides a proof of concept for the definition of PD associated α-syn-specific TCRs, further studies of HLA-matched individuals with larger numbers of subjects will be required to thoroughly address diagnostic utility. Moreover, the involvement of α-syn-specific T cell responses and associated α-syn-specific TCRs can be investigated in other synucleinopathies such as multiple system atrophy (MSA) and dementia with Lewy Bodies (DLB).

The present study represents individual antigen-specific repertoires that can be considered as a sampling from the possible collective repertoire. There is limited data on similar sequences within a collective repertoire i.e., pooling of sequences from multiple individuals^19^.

It is noteworthy that clustered TCRs may be present due to an evolutionary focusing on particular epitopes, as they have primarily been described in the responses to pathogens that have coexisted with humans for long evolutionary periods, such as herpesviruses and *Mycobacterium tuberculosis*^12,17^.

To compare the specificity of TCR response in PD, we examined the TCR repertoires associated with α-syn responses to those associated with PT. PT is a foreign antigen and we measured responses against the epitopes from the antigens contained in the acellular Pertussis vaccine, from which 132 epitopes have been defined^11^. Vaccination and boosting with the TDaP vaccine (PT in combination with tetanus and diphtheria) is ubiquitous, most individuals receives multiple booster immunizations throughout life, and therefore most human subjects exhibit a vigorous T cell response to this PT peptide pool^11,20^. This is in contrast to influenza, where the vaccine composition and peptide epitopes change every year, or to other ubiquitous pathogens (such as CMV) where responsiveness is dependent on the individuals being chronically infected. Furthermore, the PT peptide pool is associated with HLA class II restricted CD4 responses, like α-syn, and it has been extensively characterized^11,20–23^. α-Syn, in contrast, is a small self-antigen with only 11 defined T cell epitopes, and so we expected to find a much narrower repertoire for α-syn. Surprisingly, we found that the TCR repertoire for α-syn-specific T cells is as restricted as that of PT-specific T cells, and concomitantly we found no difference in the PT-specific TCR repertoire between PD and HC. α-Syn brain pathology is quite common in older adults and it is difficult to know whether a HC will go on to develop PD in the future. For this reason, we did not compare α-syn-specific responses between the cohorts, instead focusing on α-syn-specific clonotypes in comparison with PT. The result that the α-syn and PT repertoire are similar is surprising, given the inherent differences in terms of nature of exposure, and number of epitopes available. It should be noted that the size of the PT repertoire is likely a reflection of repeated vaccine boosting and multiple re-exposures over an individual’s lifespan. It is possible that the overall similar complexity of TCRs might reflect repeated (in the case of PT), or chronic (in the case of α-syn) exposure and stimulation, progressively narrowing the repertoire to a similar degree.

**Figure 5:**
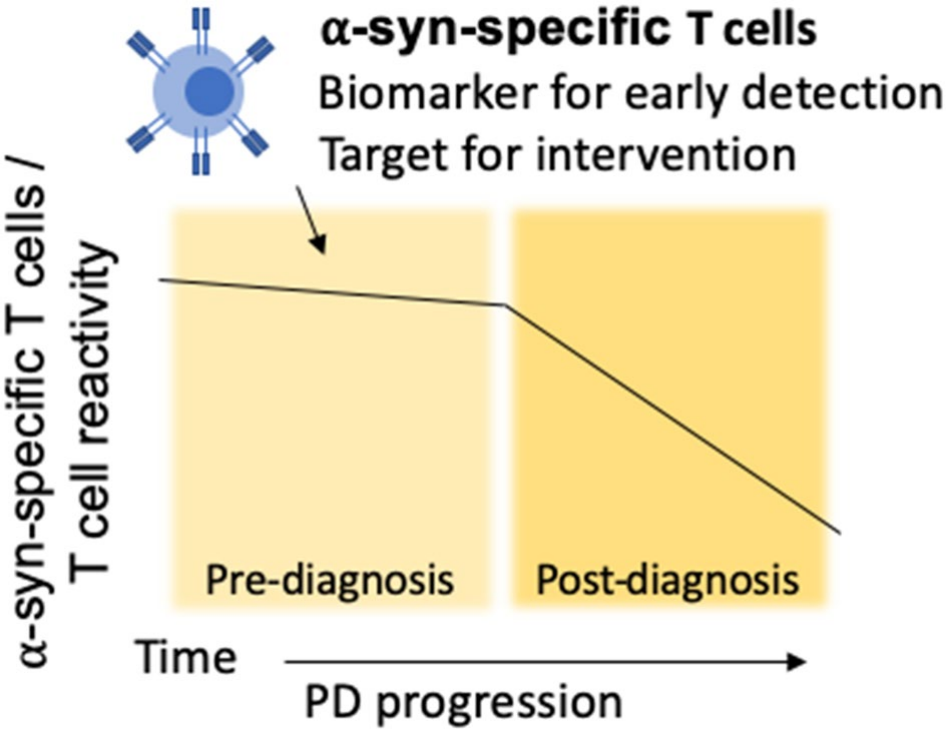
The potential role of α-syn-specific T cells in PD pathogenesis. α-syn-specific T cell reactivity decrease as PD progresses^10^, with the highest reactivity present pre-diagnosis of motor PD. α-syn-specific T cells can potentially be used as a biomarker for early detection and/or if they actively participate in the disease process as targets for early intervention.

In terms of the potential for adoptive therapy, it has been hypothesized that α-syn TCRs can be cloned into “suppressive” IL-10 producing T cells, thus providing a counter balance to proinflammatory responses^24^. Our study suggests that this is conceptually possible, but also highlights potential challenges arising from antigenic and HLA diversity. It remains to determined whether T cells are actively participating in the disease process or if they are merely responding to the death of neurons that release α-syn when they die. We hypothesize that α-syn-specific T cells (or the specific TCRs) can potentially be used as a biomarker for early detection of PD (Fig. 5), and thereby earlier intervention. Furthermore, if it is proven that the T cells actively participate in the disease process and are involved in the neurodegeneration, we hypothesize that immunomodulatory interventions can be used to modify the specific T cell responses (Fig. 5).

In conclusion, this study provides a first characterization of α-syn-specific TCR clonotypes in individuals with PD. It is possible that further research that matches PD subjects of specific HLA alleles with antigen-specific TCRs, could provide as immunotherapeutics, diagnostics, and means to track longitudinal changes in the specific T cells and disease progression.

## Methods

### Ethics statement

All participants provided written informed consent for participation in the study. Ethical approval was obtained from the Institutional review boards at La Jolla Institute for Immunology (LJI; protocol numbers VD-118 and VD-124), University of Alabama (UAB; protocol number IRB-300001297), and Columbia University Medical Center (CUMC; protocol number IRB-AAAQ9714). All research was performed in accordance with relevant guidelines and regulations.

### Study subjects

We recruited a total of 20 participants with PD from the UAB Movement Disorders Clinic in Birmingham, Alabama and 55 age-matched healthy controls (HC) from the UAB Movement Disorders Clinic (n = 10), the Alzheimer’s disease research center at CUMC (n = 25), and Precision Med, a Contract Research Organization (n = 20). Cohort characteristics of PD and HC are listed in Table 1. Blood samples were collected by trained staff. The PD cohort was recruited from the clinical practice of the UAB Movement Disorders Clinic. Participants were enrolled by fellowship trained movement disorders specialists who identified subjects using the UK Parkinson’s Disease Society Brain Bank criteria for PD. All subjects had clinically moderate to advanced PD; 2 of three cardinal signs (rest tremor, rigidity, and/or bradykinesia); age at PD diagnosis 47–75, clear clinical evidence of dopaminergic medication benefit, age at enrollment 48–90, and ability to provide informed consent. The exclusion criteria included: atypical or secondary (i.e. medication-induced) parkinsonism or other neurological disorders; history of cancer within the past 3 years (except skin), known autoimmune disease (except thyroid), or chronic immune-modulatory therapy. Age-matched HC were selected on the basis of age and ability to provide informed consent. Exclusion criteria were the same as for PD donors, and in addition we excluded self-reported family history of PD in a first-degree relative.

The selection of PD participants for the TCR repertoire analysis was based on their response to α-syn and having sufficient numbers of available cells. The healthy controls were selected to be as closely matched to the PD cohort as possible i.e., recruited at the same site, same age and sex.

### Peptides

Peptides were synthesized by A&A, LLC (San Diego, CA) as purified material (> 95% by reversed phase HPLC). α-Syn peptides were eleven 15-mers previously described as T cell epitopes^9^, which were combined into one α-syn peptide pool^10^. These peptides represent the epitopes that were most frequently recognized by individuals with PD^9^. PT peptides were 132 16-mers derived from *B. pertussis* antigens included in the aP vaccines (FHA, FIM2/3, PRN, and PT) from the Tohama I and 18,323 strains^11,20^. These were previously described as T cell epitopes recognized by more than 5% of wP or aP vaccinated subjects^11^. The PT peptides were used as one peptide pool. Peptides were aliquoted in small volumes and stored at − 20 °C to avoid multiple freeze–thaw cycles.

### PBMC isolation and in vitro expansion

Venous blood was collected in anticoagulant (e.g. heparin or EDTA)-containing blood bags or tubes. Peripheral blood mononuclear cells (PBMC) were purified from whole blood using Ficoll-PaqueTM density-gradient centrifugation, according to the manufacturer’s instructions (GE Healthcare Bio-Sciences, Pittsburgh, PA). Cells were suspended in FBS containing 10% (vol/vol) DMSO and cryopreserved in liquid nitrogen. For in vitro expansion, cryopreserved PBMCs were thawed in RPMI supplemented with 5% human serum (Gemini Bio-Products, West Sacramento, CA), 1% Glutamax (Gibco, Waltham, MA), 1% penicillin/streptomycin (Omega Scientific, Tarzana, CA) and 50 U/ml Benzonase (Millipore Sigma, Burlington, MA) as previously described^10^. The cells were then washed and viability was evaluated using trypan blue dye exclusion. Briefly, at a density of 2 × 10^6^ per mL, the cells were plated in each well of a 24 well plate in the presence of α-syn (5 μg/ml) or PT (2 μg/ml) peptide pool and were incubated in a 37 °C humidified CO_2_ incubator for 2 weeks. Every 3–4 days, cells were supplied with 10 U/ml recombinant human IL-2.

After 14 days of culture, cells were harvested and used for Fluorospot assay (below) and TCR sequencing. Cells for TCR sequencing were pelleted and frozen at − 80 °C until further processing. The Fluorospot assay measured cytokine-specific reactivity against the different peptide pools. For TCR sequencing, the expanded cells were all harvested and pelleted, and so cells that may produce cytokines other than the ones measured in the Fluorospot assay were included as well.

### Fluorospot assay

After 14 days of culture with α-syn or PT peptide pool, α-syn and PT-specific cellular responses were measured by IFNγ, IL-5 and IL-10 Fluorospot assay with all antibodies and reagents from Mabtech (Nacka Strand, Sweden), as previously described^10^. Plates were coated overnight at 4 °C with an antibody mixture containing mouse anti-human IFNγ (clone 1-D1K), mouse anti-human IL-5 (clone TRFK5) and mouse anti-human IL-10 (clone 9D7). Briefly, 1 × 10^5^ cells were added to each well of pre-coated Immobilon-FL PVDF 96-well plates (Mabtech) in the presence of 5 μg/ml peptide pool and incubated at 37 °C in humidified CO_2_ incubator for 20–24 h. Cells from the in vitro culture stimulated with DMSO (corresponding to the percent DMSO in the peptide pools) were used to assess non-specific/background cytokine production and PHA stimulation at 10 μg/ml was used as a positive control. All conditions were tested in triplicates. Fluorospot plates were developed according to manufacturer’s instructions (Mabtech). Briefly, cells were removed and plates were washed 6 times with 200 μl PBS/0.05% Tween 20 using an automated plate washer. After washing, 100 μl of antibody mixture containing anti-IFNγ (7-B6-1-FS-BAM), IL-5 (5A10-WASP), and IL-10 (12G8-biotin) prepared in PBS with 0.1% BSA was added to each well and plates were incubated for 2 h at room temperature. Plates were again washed 6 times with 200 μl PBS/0.05% Tween 20 using an automated plate washer and incubated with diluted fluorophores (anti-BAM-490, anti-WASP-640 and SA-550) for 1 h at room temperature. Finally, plates were once more washed 6 times with 200 μl PBS/0.05% Tween 20 using an automated plate washer and incubated with fluorescence enhancer for 15 min at room temperature. The plates were blotted dry and spots were counted by computer-assisted image analysis (AID iSpot, Aid Diagnostica GMBH, Strassberg, Germany). Responses were considered positive if the net spot-forming cells (SFC) per 10^6^ PBMC were ≥ 100, the stimulation index ≥ 2, and *p* ≤ 0.05 by Student’s t-test or Poisson distribution test.

### Isolation of CD4 T cells

On day one when PBMCs were thawed, CD4^+^ T cells were isolated by negative selection using the CD4 purification T cell isolation kit II (Miltenyi Biotec, Bergisch Gladbach, Germany) according to manufacturer’s instructions. Briefly, PBMCs were incubated together with the biotin-antibody cocktail for 10 min at 4 °C and then with anti-biotin microbeads for 15 min at 4 °C. Unlabeled CD4^+^ T cells were allowed to pass through the magnetic separation column. They were washed, pelleted, frozen and stored at − 80 °C until further processing.

### HLA typing

Participants were HLA typed by an ASHI-accredited laboratory at Murdoch University (Institute for Immunology & Infectious Diseases, Western Australia) as previously described^10^. HLA typing for class I (HLA A; B; C) and class II (DQA1; DQB1, DRB1 3,4,5; DPB1) was performed using locus-specific PCR amplification of genomic DNA. Patient-specific, barcoded primers were used for amplification. Amplified products were quantitated and pooled by subject and up to 48 subjects were pooled. An indexed (8 indexed MiSeq runs) library was then quantitated using Kappa universal QPCR library quantification kits. Sequencing was performed using an Illumina MiSeq using 2 × 300 paired-end chemistry. Reads were quality-filtered and passed through a proprietary allele calling algorithm and analysis pipeline using the latest IMGT HLA allele database as a reference. The algorithm was developed by E.P. and S.M. and relies on periodically updated versions of the freely available international immunogenetics information system (http://www.imgt.org) and an ASHI-accredited HLA allele caller software pipeline, IIID HLA Analysis suite (http://www.iiid.com.au/laboratory-testing/).

### TCR sequencing

DNA was extracted from the cultured cells or ex vivo CD4^+^ T cell samples using DNeasy Blood and Tissue kit (Qiagen, Hilden, Germany) according to manufacturer’s instructions. Samples were sent to Adaptive Biotechnologies (Seattle, WA) for TCRB sequencing according to their protocol. The ex vivo CD4^+^ T cell samples were sequenced with “deep resolution” to cover a maximum number of clonotypes in the repertoire. Samples that were stimulated with peptide pools for 14 days and then harvested were sequenced with “survey resolution”. The identified CDR3 regions can be found in Dataset 1.

### Data analysis

Pre-processing and quality control of the raw data was performed using the immunoSEQ analyzer (Adaptive Biotechnologies, Inc.). Measurement metrics of processed data were exported in the tsv file format and downstream data analysis was performed in Python v3.7.2 and in R v3.6.3. Only the productive rearrangements and the corresponding productive templates were considered for analysis. To identify clonotypes that were expanded in culture, each of the culture replicates for every donor was compared to the corresponding ex vivo CD4 sample. *p* values and odds-ratios were calculated using a two-sided Fisher exact test, using the ‘fisher exact’ function in the SciPy v1.3.0^25^ and NumPy v1.16.1^26^ extensions of Python. Clonotypes were considered significant if the -log_2_ odds ratios (OR) > 1 or < -1 and the false discovery rate (FDR) *p* value < 0.05, correcting for multiple testing using the Benjamini–Hochberg method^27^, and calculated using the ‘fdrcorrection’ function from the statsmodels module v0.9.0^28^ for Python. For visualization purposes, all −log_10_ FDR *p *values > 50 were set to 50. Sequence similarity by clustering was performed using GLIPH v1.0 with default parameters in order to identify conserved motifs and the global similarity of complementarity-determining region 3 (CDR3) sequences^12^.

## Supplementary Information

Supplementary Information 1.

Supplementary Information 2.
