# Supplementary material for: The TCR repertoire of α-synuclein-specific T cells in Parkinson’s disease is surprisingly diverse
Source: Sci Rep. 2021 Jan 11;11:302. doi: 10.1038/s41598-020-79726-9 (PMC7801678; doi:10.1038/s41598-020-79726-9)
Supplement: Supplementary file 2 — Supplementary Information 2. [file 41598_2020_79726_MOESM2_ESM.pdf]

The TCR repertoire of α-synuclein-specific T cells in Parkinson's disease is surprisingly diverse

Akul Singhania, John Pham, Rekha Dhanwani, April Frazier, Juliana Rezende Dutra, Karen S. Marder, Elizabeth Phillips, Simon Mallal, Amy W. Amara, David G. Standaert, David Sulzer, Bjoern Peters, Alessandro Sette, Cecilia S. Lindestam Arlehamn

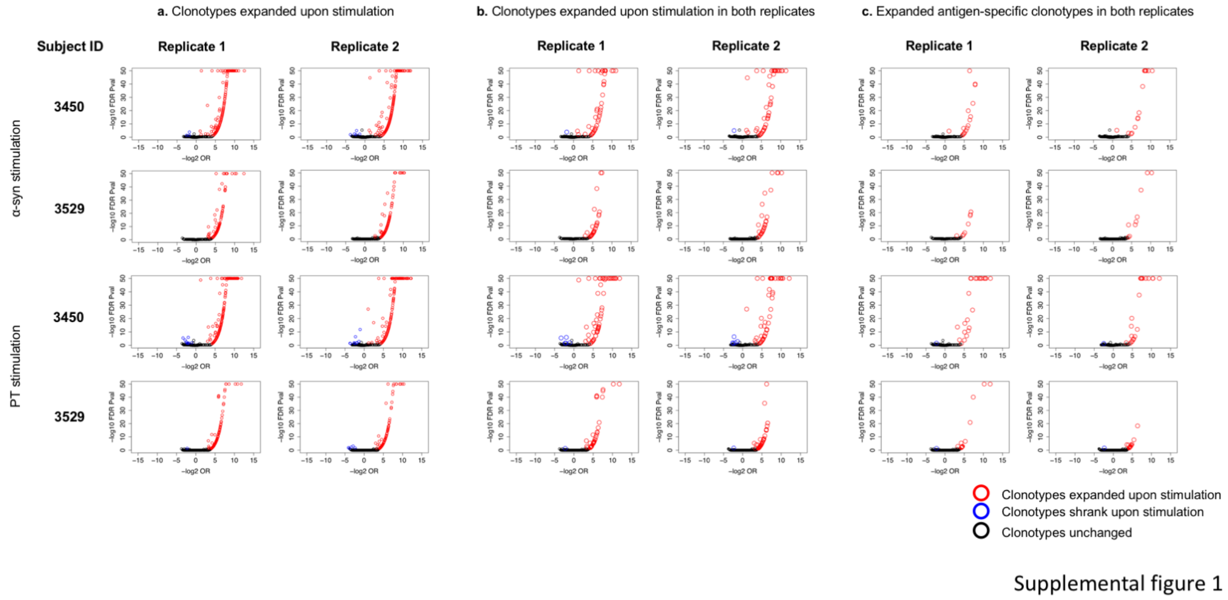

Supplementary Figure 1. Specific clonotypes expand in response to α-syn and PT peptides. (a) Clonotypes expanded in response to antigen stimulation in each replicate. (b) Clonotypes that have expanded in both replicates in response to antigen with their magnitude of expansion in both replicates represented. (c) Antigen-specific clonotypes expanded in both replicates, excluding clonotypes that also expand in response to the other antigen. Plots for two representative PD subjects are shown. α-Syn, top two rows, PT, bottom two rows. Expanded clonotypes following stimulation in red and clonotypes present at higher frequency before stimulation in CD4 T cells in blue. Black indicates clonotypes that did not expand.

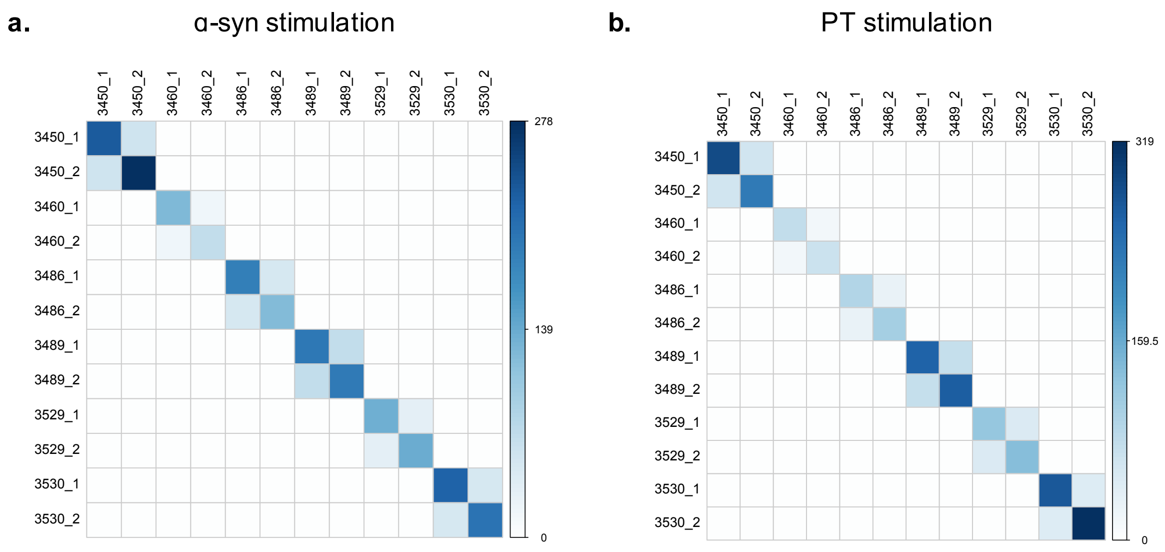

Supplementary Figure 2. Individual-specific repertoire in PD patients. Overlap of the significantly expanded clonotypes between donors and replicates upon (a) α-syn, or (b) PT stimulation, based on the exact clonotype rearrangement sequence. Blue indicates shared clonotypes, with higher color intensity indicating greater overlap, and white indicates no overlap.

Supplementary Table 1. GLIPH analysis

	Subject ID	Clonotypes expanded in			GLIPH analysis				
		Replicate 1	Replicate 2	Both replicates	3-mers	4-mers	Convergence groups[1]<br>No. groups<br>(No. peptides)	Global convergences	Local convergences
Similarity between Replicate 1 and 2 after α-syn stimulation within each PD subject	3450	231	278	56	1	1	3 (4)<br>4 (2)	7	12
	3460	124	67	19	-	-	-	-	-
	3486	190	122	48	-	-	3 (2)	3	-
	3489	199	197	67	-	1	1 (4)<br>3 (2)	4	6
	3529	136	137	33	-	-	2 (2)	2	-
	3530	225	204	49	-	1	1 (4)<br>3 (2)	2	28
Similarity between clonotypes expanded in both replicates across all subjects					-	-	-	2	-
Similarity between Replicate 1 and 2 after PT stimulation within each PD subject	3450	287	226	61	-	1	1 (5)<br>7 (2)	7	10
	3460	78	70	17	1	-	1 (4)	-	6
	3486	93	108	30	-	-	1 (2)	1	-
	3489	258	264	75	-	-	1 (2)	1	-
	3529	122	135	48	-	-	1 (2)	1	-
	3530	269	319	47	-	2	2 (4)<br>7 (2)	8	12
	Similarity between clonotypes expanded in both replicates across all subjects				-	-	2 (2)	2	-
Similarity between Replicate 1 and 2 after PT stimulation within each HC subject	3447	135	134	56	-	-	1 (2)	1	-
	3452	270	239	45	2	1	1 (5)<br>3 (4)<br>3 (2)	13	18
	3457	299	363	121	-	2	1 (5)<br>1 (4)<br>4 (2)	5	12
	3459	194	143	78	-	-	3 (2)	3	-
	3466	241	221	112	-	2	1 (5)<br>1 (4)<br>2 (2)	5	16
	3487	123	127	50	-	-	2 (2)	2	-
	3495	122	182	37	-	1	1 (6)<br>3 (2)	9	6
	Similarity between clonotypes expanded in both replicates across all subjects				-	1	1 (4)<br>8 (2)	8	6
	Similarity between clonotypes expanded in both replicates across all subjects in PT-stimulated conditions (PD and HC)				-	-	2(3)<br>10 (2)	14	-

[1] Convergence groups are reported as the number of groups with the number of peptides in each.

Supplementary Table 2. HLA-type of the participants that were studied by TCR sequencing.

ID	A	B	C	DPB1	DQA1	DQB1	DRB1	DRB3/4/5
3450	A*01:01/ A*03:01	B*07:02/ B*08:01	C*07:01/ C*07:02	DPB1*04:01/ DPB1*04:01	DQA1*01:01/ DQA1*01:02	DQB1*05:01/ DQB1*06:02	DRB1*01:01/ DRB1*15:01	DRB5*01:01/
3460	A*01:01/ A*03:01	B*07:02/ B*08:01	C*07:01/ C*07:02	DPB1*02:01/ DPB1*04:01	DQA1*01:02/ DQA1*05:01	DQB1*02:01/ DQB1*06:02	DRB1*03:01/ DRB1*15:01	DRB3*01:01/ DRB5*01:01
3486	A*02:01/ A*02:01	B*15:01/ B*27:05	C*03:04/ C*16:02	DPB1*04:01/ DPB1*04:02	DQA1*03:01/ DQA1*05:01	DQB1*03:01/ DQB1*03:02	DRB1*01:03/ DRB1*04:01	DRB4*01:01/
3489	A*02:01/ A*11:01	B*35:01/ B*51:01	C*03:03/ C*04:01	DPB1*04:01/ DPB1*05:01	DQA1*01:01/ DQA1*05:01	DQB1*03:01/ DQB1*05:01	DRB1*01:01/ DRB1*12:01	DRB3*02:02/
3529	A*01:01/ A*03:01	B*07:02/ B*57:01	C*06:02/ C*07:02	DPB1*04:01/ DPB1*04:02	DQA1*01:03/ DQA1*02:01	DQB1*03:03/ DQB1*06:03	DRB1*07:01/ DRB1*13:01	DRB3*01:01/ DRB4*01:01
3530	A*02:11/ A*24:02	B*15:01/ B*40:06	C*03:03/ C*15:02	DPB1*04:01/ DPB1*04:01	DQA1*01:01/ DQA1*01:03	DQB1*05:03/ DQB1*06:01	DRB1*14:04/ DRB1*15:01	DRB3*02:02/ DRB5*01:01
3447	A*02:01/ A*24:02	B*07:02/ B*40:01	C*03:04/ C*07:02	DPB1*11:01/ DPB1*15:01	DQA1*01:02/ DQA1*02:01	DQB1*02:02/ DQB1*06:04	DRB1*07:01/ DRB1*13:02	DRB3*03:01/ DRB4*01:01
3452	A*02:01/ A*03:01	B*27:05/ B*40:01	C*02:02/ C*03:04	DPB1*03:01/ DPB1*05:01	DQA1*01:02/ DQA1*01:02	DQB1*06:02/ DQB1*06:04	DRB1*13:02/ DRB1*15:01	DRB3*03:01/ DRB5*01:01
3457	A*01:01/ A*74:03	B*08:01/ B*44:03	C*04:01/ C*07:01	DPB1*02:01/ DPB1*04:01	DQA1*03:01/ DQA1*05:01	DQB1*02:01/ DQB1*03:01	DRB1*03:01/ DRB1*04:01	DRB3*01:01/ DRB4*01:01
3459	A*02:01/ A*11:01	B*08:01/ B*15:01	C*03:03/ C*07:02	DPB1*02:01/ DPB1*04:01	DQA1*01:03/ DQA1*05:01	DQB1*02:01/ DQB1*06:03	DRB1*03:01/ DRB1*13:01	DRB3*01:01/ DRB3*02:02
3466	A*03:01/ A*23:01	B*41:02/ B*49:01	C*07:01/ C*17:01	DPB1*03:01/ DPB1*04:01	DQA1*01:02/ DQA1*05:01	DQB1*03:01/ DQB1*05:02	DRB1*13:03/ DRB1*16:01	DRB3*01:01/ DRB5*02:02
3487	A*11:01/ A*30:01	B*08:01/ B*51:01	C*07:01/ C*14:02	DPB1*01:01/ DPB1*03:01	DQA1*01:03/ DQA1*05:01	DQB1*02:01/ DQB1*06:03	DRB1*03:01/ DRB1*13:01	DRB3*01:01/ DRB3*01:01
3495	A*01:01/ A*24:02	B*08:01/ B*40:01	C*03:04/ C*07:01	DPB1*04:01/ DPB1*04:01	DQA1*01:02/ DQA1*01:03	DQB1*06:02/ DQB1*06:03	DRB1*13:01/ DRB1*15:01	DRB3*02:02/ DRB5*01:01
